# Behavior problems and prevalence of asthma symptoms among Brazilian children

**DOI:** 10.1016/j.jpsychores.2011.02.004

**Published:** 2011-09

**Authors:** Caroline A. Feitosa, Darci N. Santos, Maria B. Barreto do Carmo, Letícia M. Santos, Carlos A.S. Teles, Laura C. Rodrigues, Mauricio L. Barreto

**Affiliations:** a Institute of Collective Health, Federal University of Bahia Salvador, Brazil; b Institute of Psychology, University of São Paulo, São Paulo, Brazil; c Institute of Statistics, State University of Feira de Santana, Feira de Santana, Brazil; d London School of Hygiene and Tropical Medicine, London, UK

**Keywords:** SCAALA, Social Changes, Asthma and Allergy in Latin America, ISAAC, International Study of Asthma and Allergies in Childhood, CBCL, Child Behavior Checklist, ICC, Intraclass correlation coefficient, Childhood asthma, Behavior problems, SCAALA Programme

## Abstract

**Objective:**

Asthma is the most common chronic disease in childhood and has been designated a public health problem due to the increase in its prevalence in recent decades, the amount of health service expenditure it absorbs and an absence of consensus about its etiology. The relationships among psychosocial factors and the occurrence, symptomatology, and severity of asthma have recently been considered. There is still controversy about the association between asthma and a child's mental health, since the pathways through which this relationship is established are complex and not well researched. This study aims to investigate whether behavior problems are associated with the prevalence of asthma symptoms in a large urban center in Latin America.

**Methods:**

It is a cross-section study of 869 children between 6 and 12 years old, residents of Salvador, Brazil. The International Study of Allergy and Asthma in Childhood (ISAAC) instrument was used to evaluate prevalence of asthma symptoms. The Child Behavior Checklist (CBCL) was employed to evaluate behavioral problems.

**Results:**

19.26% (n = 212) of the children presented symptoms of asthma. 35% were classified as having clinical behavioral problems. Poisson's robust regression model demonstrated a statistically significant association between the presence of behavioral problems and asthma symptoms occurrence (PR: 1.43; 95% CI: 1.10–1.85).

**Conclusion:**

These results suggest an association between behavioral problems and pediatric asthma, and support the inclusion of mental health care in the provision of services for asthma morbidity.

## Introduction

Asthma is considered the most common chronic childhood disease and mainly affects children resident in urban areas [1]. Asthma has been designated a serious public health problem due to the increase in its prevalence over the last two decades [2] and the associated high health service costs in admissions and hospitalizations [1,3]. Asthma has emerged as an important public health problem in Latin America and more specifically, in Brazil [2]. Observations from the early ISAAC (International Study of Asthma and Allergies in Childhood) showed that the prevalence of asthma symptoms in Latin American countries was as high as reported in the UK, Australia and New Zealand [2]. The reported prevalence of asthma in Salvador, Brazil was 24.6% among school children aged 13 and 14 years old, one of the highest in the world [4].

The cause of asthma is not yet completely understood, and there is no consensus about its etiology [5]. A vast body of research emphasizes the role of genetic and environmental factors in the appearance of asthma, and a great deal of interest has recently emerged concerning the relationship between psychosocial factors and asthma morbidity [5–8].

Many studies have examined the relationship between child mental health problems and chronic diseases, however the results have been contradictory [9–13]. Despite inconsistencies in the literature, a meta-analysis of 78 studies suggested that children who suffer from asthma, particularly severe asthma, present a greater prevalence of psychological problems when compared to non-asthmatic children [14].

The association between asthma and childhood behavioral problems has been identified, mainly through higher behavioral problem scores in asthmatic children, with significant differences found in multiple domains between groups [12,13,15]. Further, studies draw attention to the role of psychological problems in the symptomatology and severity of asthma, demonstrating that children with higher scores in behavioral problems present 18 more days of wheezing per year [7].

The co-occurrence between behavior problems and asthma has been associated with lower adherence to treatment and increased frequency and duration of hospitalizations [5,7]. In addition, the presence of childhood psychological disorders, particularly depression, was found to be a contributing factor for asthma occurrence and mortality [7,16–18].

However, most of these studies were conducted in developed countries. There is an increased need for studies regarding factors associated with asthma in Latin America since prevention strategies are still poorly understood and asthma has emerged as an important cause of morbidity and mortality in these countries [3].

This study aims to investigate whether behavioral problems are associated with the prevalence of asthma symptoms in a large urban center in Latin America.

## Methods

### Study design

This is a cross-sectional study integrated into a longitudinal study entitled Social Changes, Asthma and Allergy in Latin America (SCAALA), Salvador. The SCAALA project is made up of a set of research activities carried out in Brazil and Ecuador. In Brazil, the principal objective was to investigate the association between asthma prevalence and other allergic diseases, potential risk factors such as early exposure to infections, environmental, nutritional, immunological and psychosocial factors [3]. The study population was selected through randomized sampling, and was recruited from 24 geographical micro-regions representative of the population resident in areas of Salvador. Salvador is a large urban center in Brazil with 2,443,107 inhabitants and it is located in one of the poorest regions of the country. According to recent data from the Brazilian Institute of Geography and Statistics (IBGE, 2004), 35.76% of its population is living in poverty.

### Participants

**Fig. 1:**
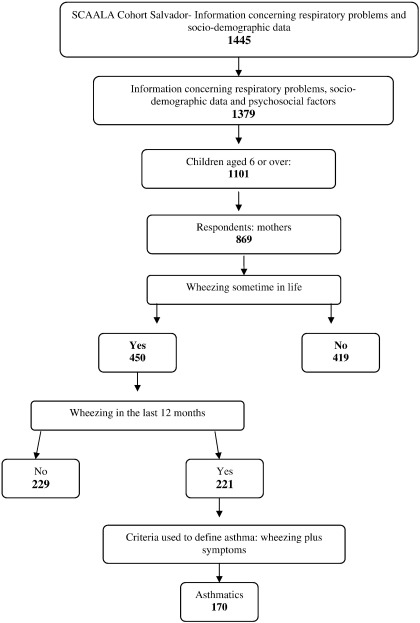
Selection of study population.

The sample was made up of 1445 children of both sexes aged 5 to 12 years old, although there was an initial loss of 79 children due to change of address or refusal to participate. For the purposes of this study, the 256 participants under 6 years old were excluded from the analysis, due to specificities in the instrument used to measure behavior problems. Moreover, only those children whose questionnaires were answered by their own mothers were included (84.1%). Thus 869 children between 6 and 12 years old were studied (Fig. 1).

### Procedures

Professionals and students of psychology, duly trained in the application of the instruments, visited the child's home between January and November 2006 in order to collect psychosocial data. Information regarding respiratory problems and socio-economic data were collected between the months of June and October 2005 by another team of appropriately trained interviewers. In both cases the mother or the child's main caregiver provided data.

### Measures

The CBCL (Child Behavior Checklist) version 2001, which provides broad coverage of the principal psychopathologic symptoms [19,20] was used to assess behavior problems. The checklist provides a total of 118 descriptions that are evaluated according to their presentation and frequency in the previous six months, using 0 as “never”, 1 as “sometimes present” and 2 as “always present”. The total value attributed to each item gives a score which is converted into a T score and whose value expresses the severity of the problem for the child studied. These problems were classified into 8 sub-scales as follows: Withdrawn, Somatic Complaints, Anxious/Depressed, Social Problems, Thought Problems, Attention Problems, Aggressive Behavior and Rule-Breaking Behavior. Another level of classification aggregates the sub-groups Withdrawn, Somatic Complaints, Anxious/Depressed into a scale evaluating problems designated ‘internalization’. The ‘externalization’ evaluation scale is made up of the following sub-groups: Aggressive Behavior and Rule-Breaking Behavior. The cut off points for total problems were calculated using T scores and categorized thus: up to 60 points as normal; between 61 and 63 points as borderline, and from 64 points as clinical for a particular problem. This cut off point is based on multicultural studies which have shown greater sensitivity in identifying clinical cases of mental ill health [19]. The CBCL was appropriately translated and validated for the Brazilian population by Bordin et al. in 1995 and this cut-off point demonstrated high sensitivity (87%), identifying 95% of the moderate cases and 100% of the severe cases [20]. For the purposes of this study, the presence of behavior problems was dichotomized; those who scored more than 64 points were considered clinical and those who scored less than this cut off point, including those within the borderline case category, normal.

The prevalence of asthma symptoms and respiratory problems was measured using a questionnaire standardized by the International Study of Asthma and Allergies in Childhood (ISAAC) for assessment of symptomatology, family history of asthma and allergic diseases, exposure to allergens, presence of smokers in the home and lifelong smoking habit [21]. This questionnaire had previously been validated for use in Brazilian Portuguese in a sample population of Brazilians [3,21]. The presence of asthma was defined according to the following criteria: reports some wheezing in the last twelve months, in addition to at least one of the following symptoms: asthma sometime in life, difficulty talking due to wheezing in the chest, waking at night due to wheezing one or more times a week and wheezing in the chest during or after physical exercise. Further analysis was carried out taking only the criterion of presentation of wheezing in the last 12 months as an outcome.

A socio-demographic questionnaire was employed to evaluate the nature of the socio-environmental context of the child through variables such as family composition, maternal schooling, family income and habits, such as smoking and drinking, basic sanitation, living conditions, and demographic characteristics.

Dust samples were collected using a residential vacuum cleaner (Eletrolux Professional, 1220 W) containing a nylon 25 μm micromesh sock filter [3]. The children's beds were aspirated over a 1 m^2^ area adjacent to the head of the bed. Filters were weighed before and after the dust sample collection and the fine particulates were weighed, aliquoted as 100 mg samples and cryo-preserved at − 20 °C. Temperature and air humidity were recorded in the bedroom with a thermohygrometer [22].

The SRQ-20 was used to assess minor psychiatric disorders in the mother. This instrument was developed by the World Health Organization [23] and validated in Brazil by Mari and Williams [24]. It is composed of 20 questions with dichotomous (yes/no) answers referring to the presence or absence of symptoms of depression, anxiety and somatic disorders in the previous month. A cut-off point for the definition of suspected cases of minor psychiatric disorders was established as 8 or more positive answers, a condition that, although not characterizing a psychiatric diagnosis, indicates significant psychic suffering. This cut-off point was defined in accordance with studies previously carried out in Brazil [24].

The potential confounders used in the study were child's sex and age, maternal schooling, monthly family income, parental asthma, minor psychiatric disorders in mother, exposure to smoke at home, alcohol misuse and presence of allergens in dust. Most of these variables have been identified in several studies as associated with both asthma and behavior problems in childhood. Studies demonstrate greater frequency of asthma in male children, especially among those aged between 4 and 5 years old [25,26]. Prevalence is also greater among children whose parents have low levels of schooling or who report asthma sometime in their lives or who, in addition, experience exposure to cigarette smoke at home [27]. Family income is considered a risk factor in first-aid asthma examinations [25]. The presence of domestic animals and allergens in domestic dust (especially mite and cockroach allergens) are factors that have also been associated with the asthmatic condition [28]. Also, several studies have consistently shown that low socioeconomic status (SES), history of psychiatric disorders among parents and maternal education are the main factors associated with the occurrence of behavior problems in childhood [29–32]. Moreover, the occurrence of psychological problems in childhood varies according to age and sex [19]. The presence of smoke in the house was found to be associated with behavioral problems in children [33].

### Statistical analysis

Data regarding socio-economic conditions, asthma and respiratory problems were double-entered using Epi Info software. Data concerning the mental health of the child, as assessed by the CBCL, were entered into the ADM Software developed by the CBCL author [34]. The STATA program version 9.0 was employed in the analysis.

A bivariate analysis was initially undertaken in order to examine differences between children with or without behavior problems using Pearson's *x*^2^ test. Following this a bivariate analysis was undertaken to estimate the prevalence of asthma according to each potential confounder using a crude prevalence ratio with a 95% confidence interval.

Variables that were considered confounding were re-tested using stratified analysis. The Intraclass Correlation Coefficient (ICC) was 9.3×10^−8^, indicating that the prevalence of asthma was not modified according to the micro-region that the child inhabited. Poisson's regression model of robust variance was applied to the multivariate analysis. Prevalence ratio was chosen as a measurement of association because it is understood that the use of logistical regression models in common events, such as asthma, tends to overestimate the measurement of association [34,35].

### Ethical considerations

Ethical approval was obtained in 2004 from the Brazilian National Ethical Committee. Written informed consent was obtained from the legal guardian of each subject. All relevant clinical results were sent to each subject's caregiver and a trained clinician made specific recommendations in each case. Children who presented with severe symptoms of mental distress or whose caregivers spontaneously requested psychological support were advised to seek help from the public mental health services.

## Results

The global prevalence of behavioral problems was 35.10% (n = 305), with the greatest percentages found in boys (36.85%), among children under 7 years old (37.02%) and in the group with the lowest family income (37.73%). High prevalence of behavior problems was also found for children whose mothers had 5 or 6 years of schooling (40.42%), whose parents reported asthma sometime in life (45.37%), who were exposed to smoke in the home (43.56%), whose mothers presented with minor psychiatric disorder (58.80%) and whose mothers use alcohol regularly (38.34%) (Table 1). A statistically significant difference was observed between the prevalence of behavioral problems according to maternal schooling, exposure to smoke in the home, minor psychiatric disorders in the mother and parental asthma (Table 1).

The total prevalence of asthma in the sample (n = 869) was 19.56% (n = 170). Bivariate analysis showed that the greatest prevalence of asthma was seen in children under 7 years old (22.84%). Similarly, the prevalence of asthma was greatest among children whose mothers had no more than 4 years of schooling (24.14%) and among mothers who presented with a family history of asthma (33.33%). Greater prevalence was also found in families with a monthly income of less than $130 dollars (21.36%) and in the group of children whose mothers presented with minor psychiatric disorders (26.24%) (Table 2).

There was no statistically significant difference between the prevalence of asthma in girls or boys, or among children who experienced or did not experience exposure to smoke in the home. Furthermore, there was no statistically significant difference between the prevalence of asthma in children whose mother drink alcohol regularly or among those exposed or not exposed to allergens in domiciliary dust (Table 2). However, when we take the criterion of wheezing in the last 12 months as an outcome, statistically significant differences were found, demonstrating that the prevalence of wheezing modifies according to age, maternal schooling, minor psychiatric disorder in the mother and parental asthma (Table 2).

Estimation of the association between behavioral problems and asthma resulted in a prevalence ratio of 1.53 (95% CI: 1.17–2.00). Regression using Poisson's model, adjusting by all the co-variables and by micro-area resulted in a prevalence ratio of 1.40 (95% CI: 1.08–1.81) for association between behavioral problems and asthma (Table 3). Similarly, estimation of the association between behavioral problems and wheezing in the last 12 months resulted in a crude prevalence ratio of 1.56 (95% CI: 1.24–1.95) and prevalence ratio of 1.48 (95% CI: 1.12–1.92) when adjusted by all co-variables and micro-area (Table 3).

In order to minimize possible bias due to somatic issues associated with the appearance and treatment of asthma, the analysis was undertaken once again removing those CBCL's items relating to the Somatic Complaints sub-scale [7,13]. There were no changes in the results (data not shown).

## Discussion

We found a global prevalence of asthma of 19.56% in the universe studied, which was greater among children who presented positive for behavioral problems (25.25%) compared to the group of children who did not (16.49%). These prevalence ratios (PR: 1.40; CI 95%: 1.08–1.81) suggest that the group of children who tested positive for behavioral problems present a 40% greater occurrence of asthma in relation to the group of children who did not. This ratio remains statistically significant after adjusting for confounding variables. The global prevalence of wheezing in the last 12 months was 25.4%, rising to 33.11% in children with behavioral problems, while for children without behavioral problems the percentage was 21.28%.

The association between behavioral problems and asthma found in our study is maintained independently of the morbidity criterion adopted as a definition of outcome, demonstrating that the prevalence produced for both was greater among children who presented behavioral problems when compared to those without such problems. These results are in line with the literature on the relationship between child mental health and asthma, which reports greater prevalence of behavioral problems in asthmatic children when compared to groups of non-asthmatic children [7,12,16]. However, most of these studies were conducted in developed countries and we must emphasize that this study was carried out in a population representative of poor populations living in a large urban center in Latin America.

The study of Weil et al. [7] reports an increase in asthma severity associated with behavioral problems. One possible explanation for these results is that living with behavioral problems in childhood may affect the management of asthma symptoms and suggests difficulty in following treatment, which thus increases functional impairment and the severity of the disease.

Bearing in mind the complexity of the factors studied and the lack of consensus concerning their etiology, the study design employed here does not allow us to establish a causal direction in the relationship between asthma and behavioral problems. However, recent longitudinal studies suggest that behavioral problems precede the appearance of asthma symptoms [7,16]. On the other hand, only one cohort study assessed the impact of asthma on internalization problems, finding a positive relationship [36], and one other assessed the two possible lines of causality [13].

While accepting that living with asthma morbidity is a risk factor for behavioral adjustment problems, McQuaid [14] asserts that this relationship is of a bidirectional, even circular, nature. However, Ortega et al's study [6] suggests that understanding the relationship between child mental health and asthma requires an integrated model incorporating environmental factors, parental mental health and psychological problems in the child.

Studies conducted in Latin America have reported highly variable prevalence rates of behavior problems in children. This is could be attributable to a lack of methodological uniformity; studies sample size, measurement and evaluation tools differ widely [37]. Despite this heterogeneity, Fleitlich and Goodman, 2001 [30] reported a 35% prevalence of psychological problems in children form low SES in a cross sectional survey settled in Brazil, which is consistent with our findings. There are several contextual factors that might contribute to this high prevalence rate: the families in our sample are living in the outskirts of the city in neighborhoods which are predominantly characterized by poverty and children from low socioeconomic status (SES) reported a higher incidence of behavior problems as compared to the general population [30,32]. Also, history of psychiatric disorders among parents, has been associated with the occurrence of behavior problems in childhood [29–31], and the overall prevalence of mental disorders among female caregivers in the same area, assessed according to the CIDI version 2.1 has been found to be 47.5% (95% CI 0.42–0.53) [38]. Other possible explanation for the high prevalence is the lack of access to psychological services. Kataoka et al., 2002 [39] found that children with low SES and from Latin and black backgrounds were more likely to not attend services of psychological care, although the need for mental service in these populations was high. In Brazil there is a visible historical gap in public mental health care for children and adolescents [40].

It is important to emphasize that all study variables were assessed via maternal report. Although there are potential limitations involving this strategy, we considered these standardized questionnaires to be reliable measures since both CBCL and ISAAC were developed and validated to access psychological and asthma symptoms in children under 11 years via parental report [19,21]. Moreover, the study of validation of the ISAAC questionnaire in Brazil found a significant agreement between the adolescents' responses to the asthma questionnaire and those from their parents (74.3%) [21]. Studies comparing parents' reports and adolescents' self-reports of behavior problems found that although adolescents reported more problem behaviors than their parents, there is a good degree of agreement between the two sources of information [41,42].

Another limitation of the present study is related to the collection of psychosocial data, which took place after the collection of data regarding asthma and respiratory problems. Further longitudinal studies are therefore needed in order to assess the occurrence of asthma morbidity and to clarify the probable impact of reverse causality. Independent of the direction of this association, further attention has been paid to the relationship between psychosocial aspects and the occurrence of chronic diseases, and specifically asthma, since the psychosocial environment is seen as an important factor associated with the etiology, appearance and symptomatology of this morbidity [5–7,12,16,18,43].

The results of this study could contribute to the development of potentially useful public health interventions in a developing country where there is no public strategy established to prevent asthma. An understanding of the multiple factors associated with asthma morbidity will thus lead to the inclusion of psychological measures in the planning of health and childcare services for asthmatic children [43]. Once the impact of psychosocial factors on asthma prevalence is clarified, psychologists could be included in the pediatric team, which would lead to the provision of integrated and multidisciplinary services for the management and prevention of asthma morbidity.

## Figures and Tables

**Table 1:** Behavior problems according to social demographic variables

	Behavior problems		
Variables	N	Prev. (%)	P value
Sex			
Female	134	33.09	0.246
Male	171	36.85	
Age			
< 7 years	154	37.02	0.255
> 7 years	151	33.33	
Maternal education			
< 5 years	58	33.33	0.002
5 to 8 years	173	40.42	
≥ 9 years	77	27.65	
Family income^a^			
< 130	166	37.73	0.137
> 130	134	32.84	
Smoke in the home			
Yes	98	43.56	0.002
No	207	32.14	
Parental asthma			
Yes	49	45.37	0.017
No	256	33.64	
Minor psychiatric disorders in mother			
Yes	167	58.80	0.001
No	117	22.20	
Mother drinks alcohol			
Yes	194	38.34	0.024
No	105	30.79	

a US Dollar–Brazilian Real exchange rate of February 2009.

**Table 2:** Prevalence and crude prevalence ratio of asthma and wheezing according to associated factors

Potential confounders	Asthma (wheezing+symptoms)		(Only wheezing)	
	N (%)	Crude PR (95% CI)	N (%)	Crude PR (95% CI)
Sex				
Male	464 (19.61)	1.00 (0.77–1.32)	464 (25.43)	0.99 (0.79–1.25)
Female	405 (19.51)		405 (25.43)	
Age				
< 7 years	416 (22.84)	1.38 (1.05–1.81)	416 (30.29)	1.44 (1.15–1.82)
> 7 years	453 (16.56)		453 (20.97)	
Maternal education				
< 5 years	174 (24.14)	1.34 (0.97–1.83)	174 (29.89)	1.23 (0.95–1.60)
5 to 8 years	428 (20.09)		428 (25.93)	
≥ 9 years	264 (15.91)		264 (21.97)	
Parental asthma				
Yes	108 (33.33)	1.89 (1.39–2.57)	108 (38.89)	1.65 (1.26–2.16)
No	761 (17.61)		761 (23.52)	
Family income^a^				
<$130	440 (21.36)	1.18 (0.89–1.55)	440 (28.18)	1.24 (0.98–1.56)
>$130	408 (18.14)		408 (22.79)	
Smoke in the home				
Yes	225 (19.56)	0.99 (0.73–1.36)	225 (22.67)	0.86 (0.65–1.13)
No	644 (19.57)		644 (26.40)	
Minor psychiatric disorder in mother				
Yes	289 (26.64)	1.57 (1.16–2.14)	289 (33.22)	1.47 (1.12–1.92)
No	527 (16.89)		527 (22.58)	
Alcohol use				
Yes	506 (20.36)	1.08 (0.79–1.48)	506(25.69)	1.01 (0.76–1.32)
No	341 (18.77)		341 (25.51)	
Allergens in dust				
*Dog*				
Yes	655 (19.40)	0.90 (0.66–1.24)	655 (24.51)	0.84 (0.65–1.09)
No	182 (21.43)		182 (29.12)	
*Cat*				
Yes	646 (14.93)	1.43 (0.99–2.05)	646 (26.79)	1.25 (0.93–1.68)
No	201 (21.36)		201 (21.39)	
*Dermatophagoides*				
Yes	736 (19.80)	0.99 (0.65–1.51)	736 (25.27)	0.98 (0.69–1.40)
No	101 (19.70)		101 (25.74)	
*Blatella* germanica				
Yes	209 (18.18)	0.89 (0.64–1.23)	209 (25.84)	1.01 (0.77–1.32)
No	626 (20.45)		626 (25.56)	

a US Dollar–Brazilian Real exchange rate of February 2009.

**Table 3:** Prevalence ratio of wheezing and asthma symptoms according to childhood behavioral problems

Behavior problems	N (%)	Non adjusted PR (95% CI)	Adjusted PR^a^ (95% CI)
(Wheezing in last 12 months)			
Yes	305 (33.11)	1.56 (1.24–1.95)	1.48 (1.12–1.92)
No	564 (21.28)		
Asthma (wheezing+symptoms)			
Yes	305 (25.25)	1.53 (1.17–2.00)	1.40 (1.08–1.81)
No	564 (16.49)		

a Adjusted for sex, age, maternal education, income, parental asthma, minor psychiatric disorders in the mother, alcohol use, allergens in dust and smoking.
